# Tissue-specific Transcriptome analysis reveals lignocellulose synthesis regulation in elephant grass (*Pennisetum purpureum* Schum)

**DOI:** 10.1186/s12870-020-02735-3

**Published:** 2020-11-19

**Authors:** Wenqing Zhang, Shengkui Zhang, Xianqin Lu, Can Li, Xingwang Liu, Geyu Dong, Tao Xia

**Affiliations:** 1 State Key Laboratory of Biobased Material and Green Papermaking, Jinan, China; 2 School of Bioengineering, Qilu University of Technology (Shandong Academy of Sciences), Jinan, 250353 Shandong People’s Republic of China

**Keywords:** Elephant grass (*Pennisetum purpureum* Schum.), RNA-Seq, KEGG (Kyoto encyclopedia of genes and genomes), WGCNA (weighted gene co-expression network analysis), Cellulose, Lignin

## Abstract

**Background:**

The characteristics of elephant grass, especially its stem lignocellulose, are of great significance for its quality as feed or other industrial raw materials. However, the research on lignocellulose biosynthesis pathway and key genes is limited because the genome of elephant grass has not been deciphered.

**Results:**

In this study, RNA sequencing (RNA-seq) combined with lignocellulose content analysis and cell wall morphology observation using elephant grass stems from different development stages as materials were applied to reveal the genes that regulate the synthesis of cellulose and lignin. A total of 3852 differentially expressed genes (DEGs) were identified in three periods of T1, T2, and T3 through RNA-seq analysis. Kyoto Encyclopedia of Genes and Genomes (KEGG) analysis of all DEGs showed that the two most abundant metabolic pathways were phenylpropane metabolism, starch and sucrose metabolism, which were closely related to cell wall development, hemicellulose, lignin and cellulose synthesis. Through weighted gene co-expression network analysis (WGCNA) of DEGs, a ‘blue’ module highly associated with cellulose synthesis and a ‘turquoise’ module highly correlated with lignin synthesis were exhibited. A total of 43 candidate genes were screened, of which 17 had function annotations in other species. Besides, by analyzing the content of lignocellulose in the stem tissues of elephant grass at different developmental stages and the expression levels of genes such as *CesA*, *PAL*, *CAD*, *C4H*, *COMT*, *CCoAMT*, *F5H* and *CCR*, it was found that the content of lignocellulose was related to the expression level of these structural genes.

**Conclusions:**

This study provides a basis for further understanding the molecular mechanisms of cellulose and lignin synthesis pathways of elephant grass, and offers a unique and extensive list of candidate genes for future specialized functional studies which may promote the development of high-quality elephant grass varieties with high cellulose and low lignin content.

**Supplementary Information:**

The online version contains supplementary material available at 10.1186/s12870-020-02735-3.

## Background

Fiber, which is mainly composed of three biological macromolecules of cellulose, lignin, and hemicellulose, plays an essential role in plant growth and stress responses. The formation of fibers and deposition of components strengthen special types of cells, such as fiber cells and vessel cells, which form mechanical tissue to provide structural support and protection for plant cells, and produce negative pressure gradient to protect plant cells during transpiration. Fiber formation is a complex process, which requires the coordination and balance of multiple metabolic pathways [1, 2].

Cellulose is the most essential component of fiber. In the process of cellulose synthesis, cellulose synthase (CesA) monomers form cellulose synthase complex (CSC), which catalyzes the synthesis of the dextran chain of cellulose by related substrates. Generally, there are two or more CesA proteins involved in the synthesis of cellulose. To date, *CesA* has been cloned in bread wheat (*Triticum aestivum*, L.), *Arabidopsis thaliana*, maize (*Zea mays* L.), poplar (*Populust remuloides*) and some other plants, their function has been clarified [3–5]. In poplar, *PtrcesA2* and *PtrcesA1* were homologous to *Arabidopsis* mutants *IRX1* and *IRX3*, respectively. They were all expressed in the process of xylem secondary wall formation of poplar. It was speculated that these two genes may be related to cell wall formation [6].

In addition to cellulose synthase genes, other genes also play an important role in cellulose biosynthesis, such as *KORRIGAN*, sucrose phosphate synthase, cytoskeleton protein, lipid transfer protein, oxidized protein [7, 8]. *KORRIGAN* encodes 1,4-β-D-glucosidase, and its mutant exhibited a decrease in cellulose and an increase in pectin synthesis, which led to excessive callus formation. In addition, it has also been found that MYB transcription factor can regulate the expression level of cellulose synthase gene, thereby changing the cellulose content and stalk strength of plants. Researchers used candidate gene genetic transformation and map-based cloning technology to study the regulation mechanism of transcription factor (OsMYB103L) on cell wall synthesis in rice (*Oryza sativa* L.) [9].

Hemicellulose is another major component of the secondary wall. It can form a network with cellulose to make the cell wall more compact. The known hemicelluloses include xyloglucan, xylan, β-1,3 (1,4) -d-glucan and mannan. Although hemicellulose is a kind of heteropolysaccharide, most hemicellulose has a single skeleton. Except for xylan, all the main chains of hemicellulose are synthesized by cellulose synthase-like (CSL) [10]. Xylan also takes β- 1,4-glycosidic bond as the main chain, but it is synthesized by the related family members of glycosyltransferase (GT) family [11].

Lignin, the second abundant component in plant cell walls, mainly plays roles in increasing plant cell wall strength and stem bending resistance [1]. Lignin is a kind of complex phenolic polymer, its monomer synthesis is derived from the phenylpropane pathway and the lignin-specific pathway [12]. Lignin monomer synthase genes mainly include *PAL*, *C4H*, *C3H*, *4CL*, *COMT*, *CCoAMT*, *F5H*, *CAD* and *CCR*, etc., which have been studied in maize [13], *Arabidopsis thaliana* [14], poplar (*Populust remuloides*) [15], ryegrass (*Lolium perenne* L.) [16], switchgrass (*Panicum virgatum* L.) and other plants [17]. The content and composition of lignin can be changed when the expression levels of these genes were up-regulated or down-regulated. By down-regulating the expression of the lignin biosynthetic pathway gene *Pt4CL1* in poplar, the deposition of lignin and cellulose can be regulated in a compensatory fashion, which may contribute to metabolic flexibility and a growth advantages to sustain the long-term structural integrity of woody perennials [1, 2]. In *Arabidopsis*, NAC, MYB, and WRKY transcription factors were also found to be involved in the regulation of lignin biosynthesis.

Elephant grass (*Pennisetum purpureum* Schum.) is a perennial Poaceae C4 plant, originated in tropical Africa, and then widely distributed in tropical and subtropical climate regions of Asia, Africa and America. It is one of the highest biomass plants in the world, with plant height up to 3 ~ 5 m and annual biomass up to 4500 kg/hm^− 2^ [18]. The main biological characteristics of elephant grass are high photosynthesis, high yield, and resistance to biotic and abiotic stress. Elephant grass is not only a high-quality forage for livestock and poultry [19], an ideal plant for soil and water conservation, a high-quality paper pulp raw material, the raw material for biofuels preparation, but also an ideal lignocellulosic energy plant [20–22]. The characteristics of elephant grass, especially its stem lignocellulose, are of great significance for its quality as feed or other industrial raw materials. Improving the cellulose content and reducing the lignin content can promote the feed quality, the conversion and utilization efficiency of lignocellulose.

Elephant grass is the allotetraploid crop (A’A’BB, 2n = 4x = 28) with a complex genome. The species is primarily cross-pollinated due to its androgynous flowering behavior, resulting in high heterozygosity and broad genetic diversity which can be utilized in breeding programs. Although the elephant grass genome has not been deciphered yet, its genetic research has focused on the evaluation of genetic diversity by constructing molecular markers and fingerprints and determining genetic relationships [23–25]. Meanwhile, researchers have revealed the biosynthetic pathway of anthocyanins, and the molecular mechanism of early response to cadmium in elephant grass roots and leaves through RNA-seq analysis [26, 27]. Some progress has also been made in improving the agronomic traits of elephant grass by using traditional breeding methods based on phenotypic selection [24, 28]. Although the regulatory network of lignocellulose synthesis has been reported in other species such as *Arabidopsis thaliana*, maize, poplar and switchgrass, research in elephant grass is still scarce.

At present, RNA-seq analysis has been widely used in various plant research, such as using RNA-seq to analyze the genetic manipulation of the phenylpropane pathway in genetically modified tobacco, which provides new basic insights and prospects for crop improvement [29]. Comparative transcriptome analysis revealed the mechanism of anthocyanin biosynthesis in mulberry black (*Morus atropurpurea* Roxb.) and white (*Morus alba* L.) fruit genotypes [30]. RNA-seq analysis has also been gradually applied to the study of elephant grass [26, 27]. Therefore, the same methods were adopted to explore the important genes of lignocellulose synthesis in elephant grass in this study.

In this study, 12 samples of elephant grass at different stem development stages were used as materials for RNA-seq analysis, lignocellulose composition analysis and cell wall morphology observation. Combined with GO, KEGG, WGCNA and Q-PCR correlation analysis, the study identified some candidate genes and provided valuable genomic data for molecular mechanism of fiber formation in elephant grass. These results provided theoretical guidance for the regulation of lignocellulose synthesis in other fiber plants, and also provided important genetic resources for their genetic improvement.

## Results

### Changes of cellulose, hemicellulose and lignin content in elephant grass stem at different development stages

**Fig. 1:**
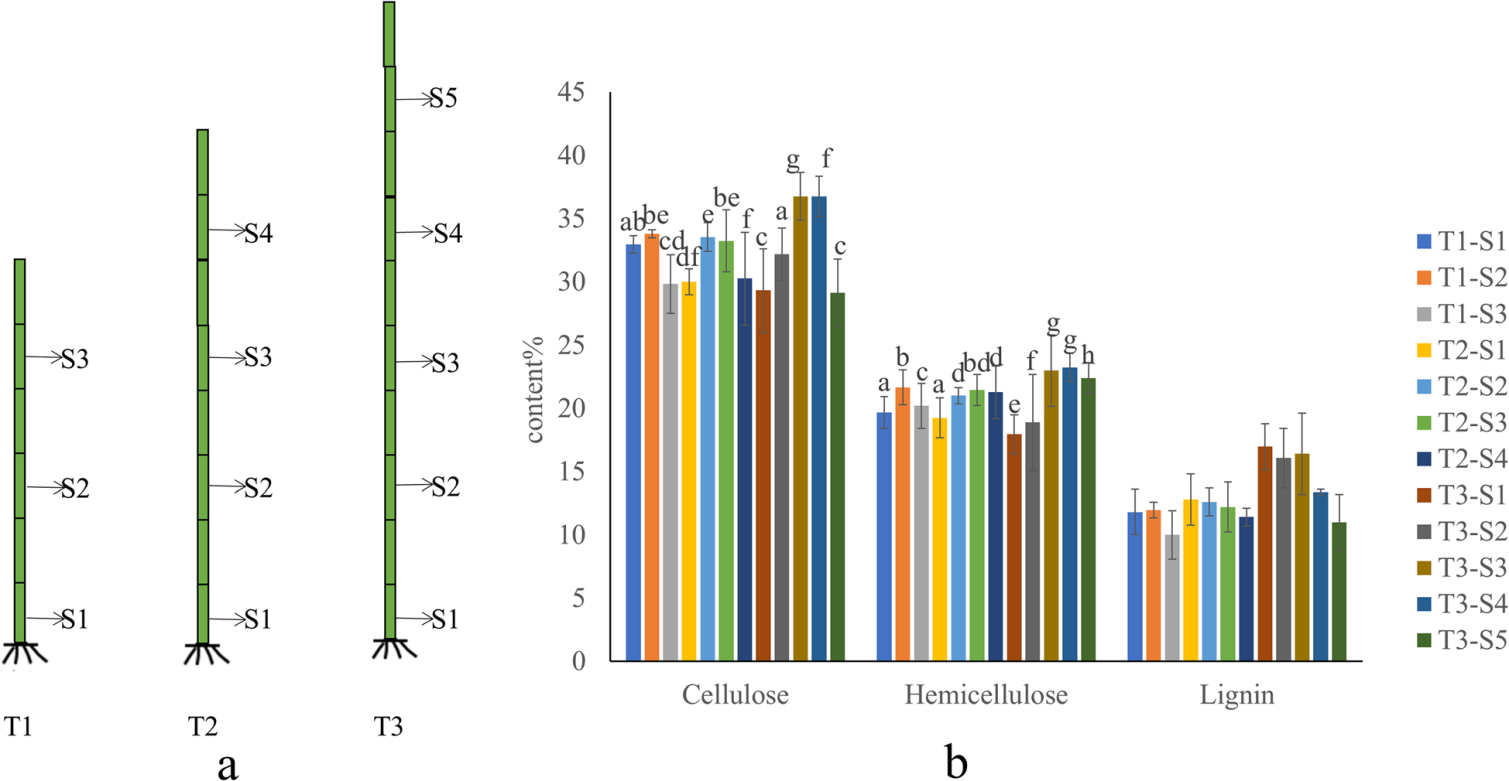
Contents of cellulose, hemicellulose and lignin at different development stages of elephant grass stems. Different letters indicate statistically significant differences (ANOVA, Duncan < 0.05). **a** Sampling period and position. **b** Cellulose, hemicellulose and lignin contents

The content of cellulose, hemicellulose and lignin in different stem segments of T1, T2, T3 phase were measured. It was found that the content of cellulose and hemicellulose increased first and then decreased. For example, the content of cellulose and hemicellulose in T1-S2 was higher than that in T1-S1, but the content of T1-S3 was lower than that of T1-S2, whereas the content of lignin fell gradually. The similar changes also appeared in different stem nodes at T2 and T3 stages. Meanwhile, by analyzing the content change of the same stem node in different development times, it was also found that with the increase of growth time of elephant grass, the content of cellulose and hemicellulose decreased, while the content of lignin increased (Fig. 1b).

By analyzing the ratio of the primary cell wall to the secondary cell wall, it was found that the secondary cell wall increases as the cell development time increases, while the rate of the cell wall to the primary cell wall keeps rising, which might be the reason of the gradually increases of lignin content to maintain and support the strength of elephant grass stems (Fig. S1).

### Characteristics of the cell wall in different developmental stages of the elephant grass stem

**Fig. 2:**
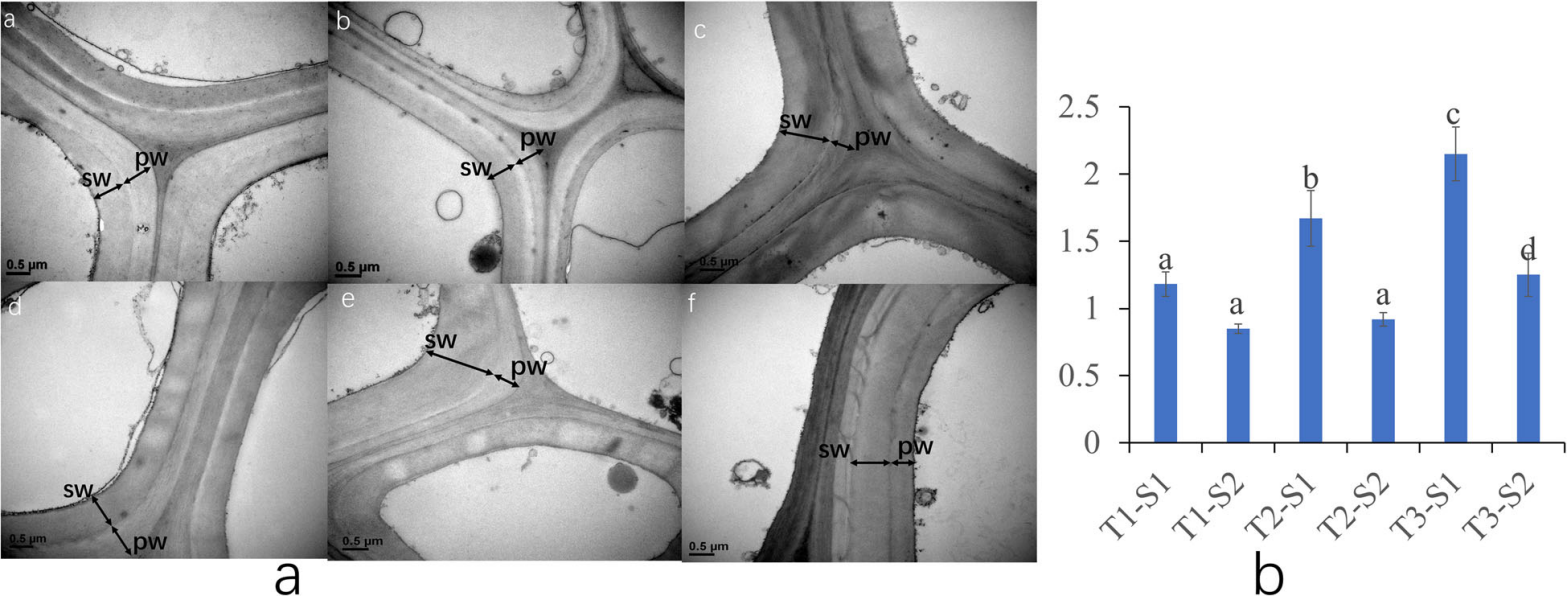
The cell wall morphology and thickness changes at different development stages of elephant grass stems. Different letters indicate statistically significant differences (ANOVA, Duncan < 0.05). **a** a.T1-S1, b. T1-S2, c. T2-S1, d. T2-S2, e. T3-S1, f. T3-S3. SW: Secondary cell wall, PW: Primary cell wall. **b** thickness ratio of secondary cell wall to primary cell wall

The changes of cell wall morphology at different development stages of elephant grass, especially the primary and secondary cell wall were observed (Fig. 2a). The ratio of secondary cell wall (sw) thickness to primary cell wall (pw) thickness in T1-S1 and T1-S2 was 1.18 and 0.85, the ratio of sw/pw thickness in T2-S1 and T2-S2 was 1.67 and 0.92, and the ratio of sw/pw thickness in T3-S1 and T3-S3 was 2.15 and 1.25, respectively (Fig. 2b). The above data showed that the change trend of sw/pw thickness of T1, T2, and T3 was consistent, that is, with the increase of development time, the development speed of secondary cell wall (sw) was faster than that of primary cell wall (pw). The ratio of sw/pw in S1 node of three different development stages was also analyzed. It was found that with the increase of development time, the thickness ratio of sw/pw in T1-S1 T2-S1, T3-S1 increased subsequently. The change of sw/pw thickness ratio in S2 and S3 stages of three different development stages showed the same trends.

To further understand the morphological changes of cell wall during the development of elephant grass, we selected S1 and S5 stem segments with a longer development time span at T3 stage as samples for micro-CT observation. The stem of elephant grass is composed of epidermis, parenchyma cells and vascular bundles. Vascular bundles, which scattered in parenchyma cells and cannot be thickened, are composed of phloem and xylem without cambium. With the development of stem tissue, S1 vascular bundle were arranged regularly and compactly (Fig. S1). The content of cellulose and hemicellulose in vascular bundle decreased gradually, while the content of lignin increased gradually (Fig. 1b) to meet the need of mechanical support during the maturation of elephant grass stems need in the process of stem maturity of elephant grass.

### Differential gene expression during the development of elephant grass stems

**Fig. 3:**
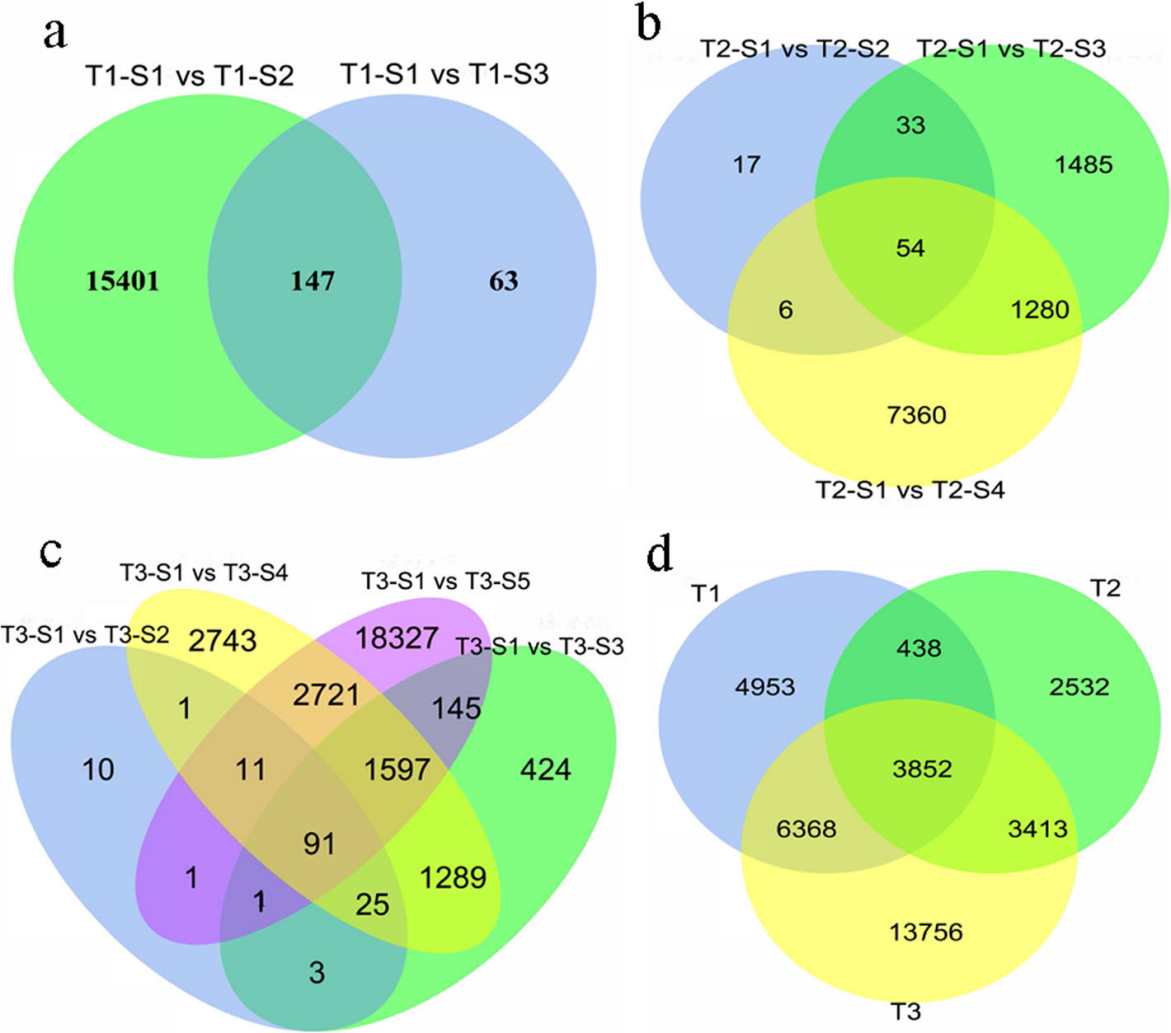
The differentially expressed genes (DEGs) identified by RNA sequence analysis in stem tissues of development stages of elephant grass. **a** The number of DEG between three adjacent stages (T1-S1, T1-S2, T1-S3). green: T1-S1 Vs T1-S2, blue: T1-S1 Vs T1-S3. **b** The number of DEG between the four adjacent stages (T2-S1, T2-S2, T2-S3, T2-S4). green: T2-S1 Vs T2-S2, blue: T2-S1 Vs T2-S3, yellow: T2-S1 Vs T2-S4. **c** The number of DEG between the five adjacent stages (T3-S1, T3-S2, T3-S3, T3-S4). Purple: T3-S1 Vs T3-S5, green: T3-S1 Vs T3-S2, blue: T3-S1 Vs T3-S3, yellow: T3-S1 Vs T3-S4. **d** DEGs between T1, T2 and T3 stages. Blue: T1 (DEGs), green: T2 (DEGs), yellow: T3 (DEGs)

Thirty-six cDNA libraries were constructed from different stages of elephant grass stems (three biological replicates for each tissue). Totally, 1.32 billion raw reads (396.96 Gb) were obtained, 1.29 billion cleaned reads (388.57Gb) were acquired after filtering with 6.56–21.07 Gb in each sample. The error rate was 0.03% (Q20 and Q30 values were more than 93 and 90%, respectively), which met the requirements of gene discovery (Table S1). De novo assembly generated 77,435 cluster sequences from 12 representative samples with the largest sequencing depth. Finally, we got a non-redundant transcript clusters include 230,572 unique genes with the average length of 961.35 bp and an N50 of 1435 bp, an N90 of 423 bp (Fig. S2). Transcriptome de novo assembly was carried out with short reads assembling program-Trinity [31]. The Pearson correlation coefficient based on the expression value of each library indicated that there was a high correlation between sample replicates (Fig. S3). Cluster analysis among samples showed that the development time of elephant grass was the main factor affecting the clustering. The DEGs in three developmental stages of elephant grass stems were analyzed, a total of 15,611, 10,235 and 27,389 DEGs were identified in T1, T2, and T3, respectively (Fig. 3). The DEGs of different stem segments at three development stages were also analyzed, it was found that 147 genes were expressed in three segments of T1, 54 in four segments of T2, and 91 in five segments of T3 (Fig. 3). The intersection of all DEGs at three different developmental stages was compared to determine the shared core set. It was found that 3852 genes were differentially expressed at three developmental stages (Fig. 3d).

**Fig. 4:**
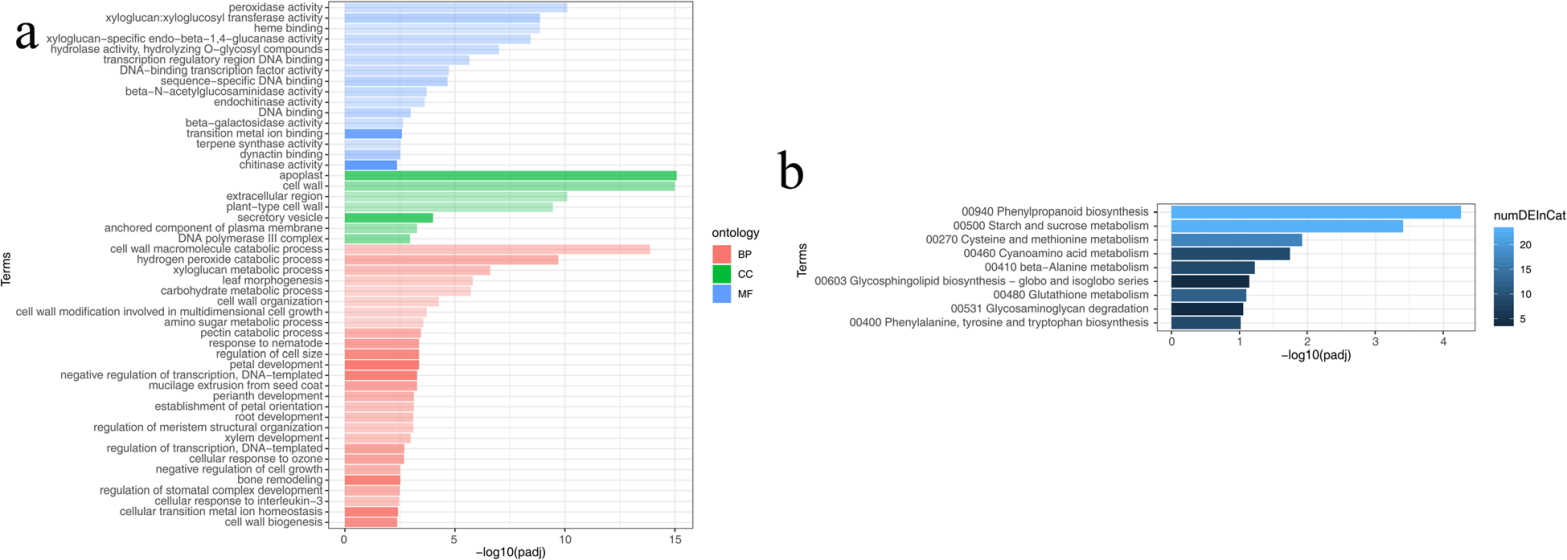
The 3852 DEGs co-expressed at three developmental stages of T1, T2, and T3 were enriched by GO and KEGG. **a** GO enrichment analysis. **b** KEGG enrichment analysis

Three thousand eight hundred fifty-two DEGs were then subjected to enrichment analysis of GO functions and KEGG pathways. 4 of the top 10 enriched GO annotation functions were related to cell composition such as apoplast, cell wall, extracellular region, plant-type cell wall, four were related to molecular functions such as peroxidase activity, xyloglucan, and xyloglucosyl transferase activity, heme-binding, xyloglucan−specific endo−beta− 1,4 − glucanase activity, and two were related to biological processes such as cell wall macromolecule catabolic process, hydrogen peroxide catabolic process (Fig. 4a) (Table S2). Based on KEGG pathway analysis, all DEGs were enriched to 9 pathways (Table S3), of which the two most significant pathways were phenylpropane metabolism (23 DEGs) and starch and sucrose metabolism (23 DEGs) (Fig. 4b).

### Genes highly correlated with the synthesis of cellulose, hemicellulose and lignin by WGCNA analysis

Weighted gene co-expression network analysis (WGCNA) was performed on 3852 DEGs at three stages, and the network was divided into three modules. The analysis of module-trait relationship showed that the ‘blue’ module was highly correlated with the synthesis of cellulose (*r* = 0.67, *P* = 6.0 × 10^− 6^) and hemicellulose (*r* = 0.51, *P* = 0.001), whereas the ‘turquoise’ module was related to lignin synthesis (*r* = 0.68, *P* = 5.0 × 10^− 6^) (Fig. S4).

**Fig. 5:**
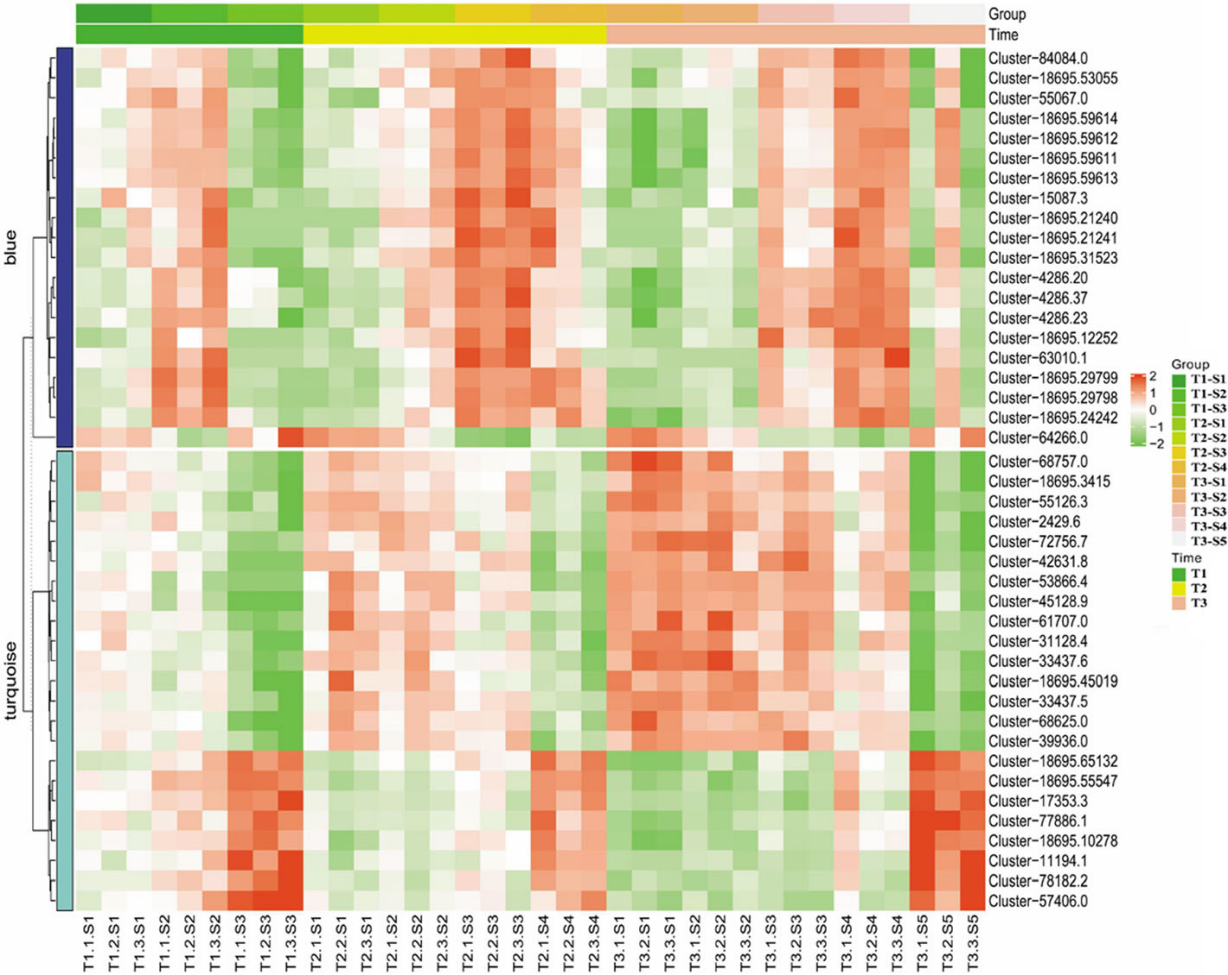
The heat maps of the expression pattern of 43 genes in WGCNA analysis at different development stages of elephant grass stems

The ‘blue’ module was filtered according to Module membership > 0.9, the absolute value of the correlation coefficient between the ‘turquoise’ module and lignin was greater than 0.75, and 20 and 23 genes remained in the ‘blue’ and ‘turquoise’ module respectively. The WGCNA gene significance (GS) (i.e., related to traits) showed that the genes with highest GS in ‘blue’ and ‘turquoise’ modules were Cluster-55,067.0 (0.659) and Cluster-17,353.3 (0.798), respectively. Six of 20 genes in ‘blue’ module were known to be functional, such as *GTL1* transcription factor, O-methyl transferase (*OMT*), expansin-likeA2, alpha-humulene synthase, probable galactinol sucrose, *GhGalT1*. Meanwhile, 14 genes with unknown functions were also covered by ‘blue’ module, which needs further study. The GO function annotations of these 20 genes in ‘blue’ module included the genes which were related to extracellular region, C-4 methyl sterol oxidase activity, terpene biosynthesis, etc. The expression levels of these genes were shown in Fig. 5.

Among 23 genes in ‘turquoise’ module, there were eight coding genes such as *GTL1*, *MYB2* transcription factors, threonine-protein kinase ERECTA, probable methionine-tRNA ligase, alcohol holding hydrogenase-like2, zinc finger protein GIS3, protein slr0074, proline-rich receptor-like protein kinase PERK8. The GO function annotations of these 23 genes in ‘turquoise’ module included genes that were related to cell growth regulation, secondary cell wall formation regulation, cell wall composition regulation, etc. 15 genes remained with unknown functions (Table S4).

### Lignin and cellulose synthesis pathway during stem development of elephant grass

**Fig. 6:**
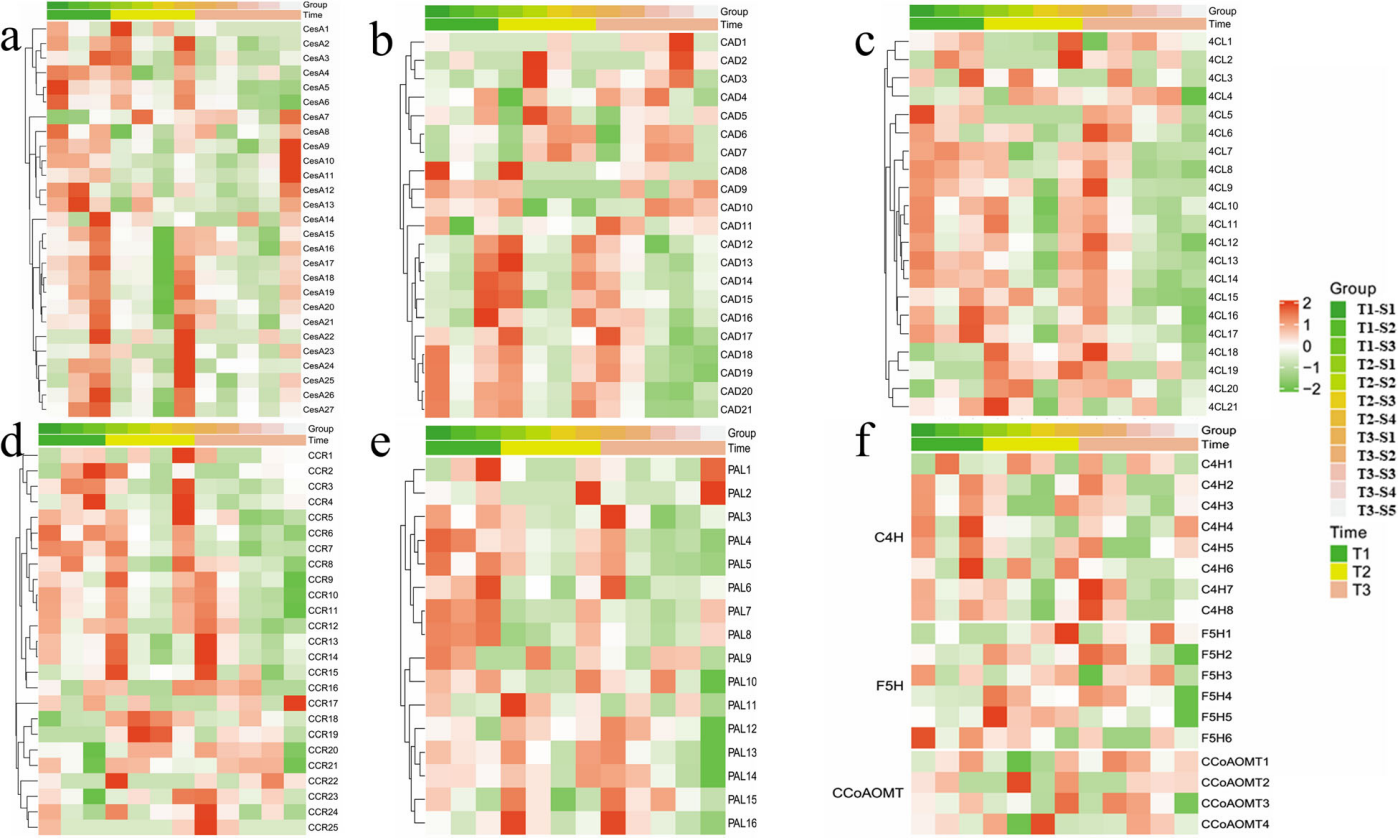
The heat maps of the expression pattern of cellulose synthase genes and lignin synthesis-related genes at different developmental stages of elephant grass stems. **a** Heat map of the expression pattern of 27 cellulose synthase genes. **b**, **c**, **d**, **e**, **f** Heat map of the expression pattern of some lignin synthesis-related genes. The grid with four different colors shows the absolute expression of genes, which are represented by different scale levels 2, 1, 0, − 1, and − 2, respectively. PAL: Phenylalanine Ammonia-Lyase; C4H: Cinnamic Acid4-Hydroxylase; CCR: Cinnamoyl-CoA Reductase; CAD: Cinnamyl Alcohol Dehydrogenase; F5H: FerulicAcid5-Hydroxylase; CoAOMT: Caffeoyl-CoAO-methytransferase; COMT: 5-Hydroxyferulic Acid O-Methyltransferase/Bispecific Caffeic Acid

Cellulose synthase (CesA) is the most important enzyme in the cellulose synthesis pathway. It can directly utilize UDPG produced by starch and sucrose metabolism to synthesize cellulose. In elephant grass, 27 *CesA* genes were identified. At T1 stage, the expression level of *CesA1* - *CesA6* was higher in the whole stem, but decreased in T2 and T3 stages, while the expression level of *CesA7* - *CesA27* in tender stems was much higher than that in mature stems at T1, T2, and T3 stages. This indicated that *CesA* gene mainly synthesizes cellulose in the tender stem tissue. When the cellulose accumulated to a certain level, its expression level gradually decreased (Fig. 6a).

Lignin synthesis is one of the most important pathways of phenylpropane metabolism. In the lignin metabolism pathway, *CAD* (21 unigenes), *4CL9* (21 unigenes), *C4H* (8 unigenes), *PAL* (16 unigenes), *CCR* (25 unigenes), *F5H* (6 unigenes), *CCoAOMT* (4 unigenes) were identified to be related. Expression analysis found that most members of these gene families showed higher expression levels in the mature stem. In contrast, a few gene members were continuously expressed in the whole tissue (Fig. 6b, c, d, e, f).

The results of qRT-PCR demonstrated that the expression trends of these genes were consistent with that of RNA-seq data (Fig. S5, Table S6). Overall, as the stem development, the expression level of cellulose synthesis genes and lignin synthesis related genes showed the opposite trend. On the other hand, the changes of stem cellulose, hemicellulose and lignin content, as well as the changes of primary stem wall and secondary cell wall thickness, were positively correlated with the expression of these two types of genes.

## Discussion

Elephant grass is one of the highest biomass forage grasses on the earth, which is widely used in feed and bioenergy related industries. It is of great significance to further understand the genes and important metabolic pathways related to lignocelluloses synthesis for molecular breeding the new elephant grass varieties and make them more suitable for industrial application.

In our study, 3852 differentially expressed genes (DEGs, adjusted *p* < 0.05) were identified in 12 stem samples of elephant grass at different developmental stages. The same method was also used to identify DEGs in the anthocyanin synthesis pathway of elephant grass and mulberry [26, 30]. The GO enrichment analysis of DEGs found that the top 10 enriched GO annotation functions included three functions that were directly related to cell wall development such as cell wall, plant-type cell wall, cell wall macromolecule catabolic process. KEGG pathway analysis discovered that sucrose and starch metabolism and phenylpropane metabolism were the most significant metabolic pathways of differential gene enrichment. Lignin synthesis is one of the important branches in phenylpropane metabolism [32], the substrate for cellulose synthesis can be provided by starch and sucrose metabolic pathways [33, 34], which indicated that these DEGs might affect the synthesis of cellulose and lignin.

In the process of cellulose synthesis, sucrose is the starting substrate, and UDPG produced by its decomposition can directly synthesize the dextran chain under the action of *CesA* gene [35]. Twenty-seven *CesA* genes were identified in elephant grass, which were more abundant than other species such as wheat (14 species) [4], *Arabidopsis thaliana* (10 species) [3], maize (10 species) [5], poplar (16 species) [8]. *CesAs* were mainly expressed in tender stem tissue (Fig. 6a). As the stem development, the cellulose content accumulated gradually (Fig. 1). The high copy of *CesAs* in elephant grass makes it has a high cellulose synthesis potential. So far, *CesA* has proven its function in breadwheat (*Triticum aestivum* L.), *Arabidopsis thaliana*, maize (*Zea mays* L.), poplar (*Populust remuloides* L.) and other plants [3–5]. The cellulose content in mature stem of elephant grass is significantly higher than that of corn, wheat, reed and other plants [36].

Totally, 101 lignin-synthesizing related genes such as *PAL*, *C4H*, *4CL*, *CCoAMT*, *F5H*, *CAD*, and *CCR* were identified in elephant grass. PAL is the critical enzyme in phenylpropane metabolism pathway, its inhibition will reduce lignin content and affect plant growth and development. 4CL is the key rate-limiting enzyme for the production of G or S-lignin monomers. Different expressions of 4CL can regulate the content of three kinds of lignin monomers, promoting or inhibiting the expression of *4CL* gene can significantly regulate the relative proportion of lignin/cellulose [37]. In elephant grass, 16 *PAL* genes and 21 *4CL* genes were mainly expressed in mature stem tissues which led to the lignin content in the mature stem of elephant grass was higher. It was also found that inhibition or overexpression of these genes in tobacco would affect the lignin content [29, 38].

To further understand the relationship between these DEGs and lignocellulose components, WGCNA was conducted and found that there were 20 genes were related to the synthesis of cellulose and hemicellulose, 23 genes were associated with the synthesis of lignin. Among these 43 genes, 14 of which have been identified with precise functions by GO annotation, some of them such as *OMT* and *GalT1* were directly related to the synthesis of lignocelluloses, some of them played important roles in growth and development and cell wall formation. There were 28 genes with unknown functions, which need further functional verification. In tobacco, it was reported that the sense or antisense expression of sequences encoding O-methyltransferase (*OMT*) could regulate enzyme activity of lignin synthesis [39]. The fiber length of transgenic cotton overexpressing *GhGalT1* was shorter than that of wild type, while in *GhGalT1* silenced line, the fiber length was significantly increased than that of wild type [40].

Generally, our work indicated the dynamic changes in cell wall composition and morphology during the stem development of elephant grass were consistent with the changes and expression of cellulose and lignin related genes. These data provided the new and extensive list of candidate genes for more specialized functional studies in the future, and an essential theoretical basis for the genetic improvements of elephant grass lignocellulose synthesis as well.

## Conclusion

RNA-seq, lignocellulose content and cell wall morphology of elephant grass stem were conducted and analyzed in this study, which provided a basis for further revealing the mechanism of lignocellulose synthesis and accumulation of elephant grass. A total of 3852 common DEGs were identified. KEGG analysis showed that the two most abundant metabolic pathways were phenylpropane metabolism (23 DEGs), starch and sucrose metabolism (23 DEGs), among which phenylpropane metabolism functioned as an important pathway for lignin synthesis, while the latter produced UDPG for cellulose synthesis. 27 *CesA* genes for cellulose synthesis and 101 related genes for lignin synthesis were identified, respectively. *CesA* genes had higher expression levels in young stems while the lignin-related genes had higher expression levels in mature stems. In addition, a total of 43 candidate genes were screened by WGCNA, of which 17 had function annotations in other species. Among them, the *GTL1* transcription factor and O-Methyl transferase (*OMT*) gene have been proved to regulate the synthesis of lignocellulose in other plant species.

## Methods

### Experimental materials

Elephant grass was cultured in the greenhouse of Qilu University of Technology, Jinan City, Shandong Province. Stalks at seedling stage 40 days (T1 period), 80 days (T2 period), 120 days (T3 period) were sampled. The lowest node of the stem was taken and labeled as S1, samples from every other stem node were labeled as S2, S3, S4 and S5 respectively. Three samples (stem nodes) were taken at T1 period (T1-S1, T1-S2, T1-S3), four samples at T2 period (T2-S1, T2-S2, T2-S3, T2-S4) and five samples at T3 period (T3-S1, T3-S2, T3-S3, T3-S4, T3-S5) (Fig. 1)a. A total of 36 samples were collected, each including three biological replicates. All samples were immediately frozen in liquid nitrogen and stored at − 80 °C before total RNA extraction.

### Determination of the content of cellulose, hemicellulose and lignin

The sample to be tested was naturally air-dried or placed in an oven to dry (temperature not exceeding 50 °C) until the moisture is less than 10%, crushed and screened by the grinder. Took a portion between 20 and 80 mesh, cooled, and stored in a sealed bag for further analysis. Content analysis was conducted by high-performance liquid chromatographic (HPLC) according to NERL method [41, 42]. The reducing sugar was analyzed by HPLC (Shimadzu, Kyoto, Japan) with a Shimadzu LC-10 AD detector. The HPLC was performed in a Bio-Rad HPX-87H column with 10 uL injected volume at 60 °C with 5 mM H_2_SO_4_ as eluent at a flow rate of 0.4 mL/min [43]. The content of cellulose, hemicellulose and lignin are the percentage of the dry weight of the sample.

### Cell wall morphology observation

The periods with the highest and lowest cellulose content at three different development stages of elephant grass stems were selected as samples, then cut the fresh stalks into 1 cm × 1 cm pieces and placed in 2.5% glutaraldehyde phosphate buffer (0.1 mol/L, pH = 7.0). Samples were treated as reported [44] and observed under EM-420 transmission electron microscope (Philips Electronics, Holland).

### Micro CT observation of elephant grass stem

The stalks of elephant grass were cut into 1 cm × 1 cm pieces and put them into 2.5% glutaraldehyde phosphate buffer (0.1 mol/L, pH = 7.0), fixed in a 4 °C storage cabinet for 3 h, washed the fixed tissue twice with 1 mol/L phosphate buffer, and placed the sample in a 40 °C oven for 24 h. Cross-section of the processed sample were observed under SkyScan 2211 micro-CT (Bruker, Belgium).

### RNA extraction and transcriptome sequencing

Total RNA was extracted from stems using HiPure Plant RNA Kit (Magen, Guangzhou, China) according to the manufacturer’s instructions. A total of 3 μg of RNA per sample was used for library preparation with insert sizes of 350 bp and sequenced on Novaseq 6000 (Illumina, USA). RNA-seq analysis was conducted using the Illumina platform according to the standard protocols [45].

### Quality control of RNA-seq and transcriptome assembly

Raw data (raw reads) of fastq format were firstly processed through Trim Galore (http://www.bioinformatics.babraham.ac.uk/projects/trim_galore/) [31]. At this step, clean data (clean reads) were obtained by removing reads containing adapter, ploy-N and low quality reads from raw data. At the same time, Q20, Q30, GC-content and sequence duplication level of the clean data were calculated. All the downstream analyses were based on clean data with high quality. 12 samples with the largest amount of sequencing were selected, assembled them with Trinity-v2.9.1 software [46], a total of 627,786 sequences were assembled, and then cd-hit-estv4.8.1 software was used to cluster 627,786 sequences based on sequence similarity of 0.97, and 477,435 representative sequences were selected [47]. The salmonv1.1.0 software was used to map the sequencing data of each sample back to 477,435 representative sequences to obtain the bam file of each sample [48]. According to the bam file, the corset software was used to cluster 477,435 representative sequences based on the comparison results of reads, and finally 230,572 representative sequences and related abundance files were obtained [49].

### Gene function annotation

Gene function was annotated based on the following databases: Nr (NCBI non-redundant protein sequences) (https://www.ncbi.nlm.nih.gov/); Swiss-Prot (A manually annotated and reviewed protein sequence database) (http://www.gpmaw.com/html/swiss-prot.html); KO (KEGG Ortholog database) (https://www.kegg.jp/); GO (Gene Ontology) (http://geneontology.org/).

### Differential expression and enrichment analysis

Differential expression analysis was performed using the R package DESeq [24]. The *p*-values were adjusted by the Benjamini-Hochberg (BH) method. Genes with an adjusted *p*-value < 0.05 were assigned as DEGs. GO enrichment and KEGG pathway enrichment analysis of 3852 differentially DEGs, which were identified in three periods of T1, T2, and T3 was implemented by R Package Goseq [50]. The population set is a set with all the annotated genes, and the study set consisted of the DEGs in the population set. The *p*-values were adjusted as differential expression analysis did. Adjusted *p*-value < 0.05 for GO enrichment analysis and adjusted *p*-value < 0.1 for KEGG pathway enrichment analysis were considered significant.

### Weighted gene co-expression network analysis

Gene expression patterns for common differentially expressed genes in T1, T2, T3 period were used to construct a co-expression network by WGCNA. The genes that were not detected to be expressed in all tissues were removed before analysis. Soft thresholds were set based on the scale-free topology criterion [51, 52].

### qRT-PCR analysis

DNase-treated RNA (2 μg) was reverse transcribed using High Capacity cDNA Reverse Transcription Kit (Applied Biosystems, Foster City, USA). Gene-specific primers were designed using Primer Express (v3.0, Applied Biosystems). Quantitative reverse transcription PCR (qRT-PCR) assays were performed using SYBR Green I Master Mix (Roche, Indianapolis, USA). Three biological and three technical replicates for each reaction were analyzed on LightCycler 480 instrument (Roche, USA) with the first step of 95 °C for 5 min followed by 40 cycles of 95 °C for 10 s, 60 °C for 10 s, and 72 °C for 20 s. Melting curves were generated using the following program: 95 °C for 15 s, 60 °C for 15 s, and 95 °C for 15 s. 18S rRNA was used as an internal control. Data analysis was calculated by 2^-ΔΔCT^ method. Significant differences between different samples were tested with IBM SPSS Statistics 19.0 software.

## Supplementary Information

**Additional file 1:** **Fig. S1.** Cell wall morphology changes of elephant grass by Micro-CT. (a) T3-S1 (b) T3-S5

**Additional file 2:** **Fig. S2.** Distribution and annotation statistics of assembled genes. (a) Length distribution and summary statistics of assembled genes. (b) In each of the three GO categories, the unigene distribution representing the most extensive level 3 gene ontology (GO). biological processes (BP), cellular components (CC) and molecular functions (MF).

**Additional file 3:** **Fig. S3.** Heat map of correlation coefficient between samples.

**Additional file 4:** **Fig. S4.** 3852 DEG sets co-expressed at three developmental stages of T1, T2 and T3 were analyzed by WGCNA.

**Additional file 5:** **Fig. S5.** RT-PCR analysis of synthetic genes related to cellulose and lignin synthesis in elephant grass stems. Different letters indicate statistically significant differences (ANOVA, Duncan < 0.05).

**Additional file 6:** **Table S1.** RNA sequencing data and corresponding quality control.

**Additional file 7:** **Table S2.** GO annotations of 43 differential genes used for WGCNA analysis

**Additional file 8:** **Table S3.** KEGG annotations of 43 differential genes used for WGCNA analysis

**Additional file 9:** **Table S4.** The expression level and functional annotation of 43 genes in WGCNA during T3 period and the correlation coefficient with lignin or cellulose synthesis.

**Additional file 10:** **Table S5.** RT-PCR primer list.

**Additional file 11:** **Table S6.** The pearson correlation analysis between RNASeq and qRT-PCR data.

## Data Availability

The datasets generated and/or analyzed during the current study are available at EBI (EMBL) project PRJEB40973 (https://www.ebi.ac.uk/ena/browser/view/PRJEB40973) with accession number ERP124692. Any reasonable requests are available from the corresponding author.

## Abbreviations

RNA-seq: RNA sequencing
DEGs: Differentially expressed genes
KEGG: Kyoto encyclopedia of genes and genomes
WGCNA: Weighted gene co-expression network analysis
CesA: Cellulose synthase
CSL: Cellulose synthase-like
GT: Glycosyltransferase
sw: Secondary cell wall
pw: Primary cell wall
GS: Gene significance
OMT: O-methyl transferase
GO: Gene ontology
KO: KEGG ortholog database
BH: Benjamini-Hochberg
UDPG: Uridine diphosphoglucose
PAL: Phenylalanine ammonia-lyase
C4H: Cinnamic acid4-hydroxylase
CCR: Cinnamoyl-coa reductase
CAD: Cinnamyl alcohol dehydrogenase
F5H: FerulicAcid5-hydroxylase
CoAOMT: Caffeoyl-coao-methytransferase
COMT: 5-Hydroxyferulic acid o-methyltransferase/bispecific caffeic acid
HPLC: High performance liquid chromatography

## References

[1] Hu WJ, Harding SA, Lung J (1999). Repression of lignin biosynthesis promotes cellulose accumulation and growth in transgenic trees. Nat Biotechnol.

[2] Zhang CB, Chen LH, Jiang J (2014). Why fine tree roots are stronger than thicker roots: The role of cellulose and lignin in relation to slope stability. Geomorphology.

[3] Samuga A, Joshi CP (2020). A new cellulose synthase gene (*PtrCesA2*) from aspen xylem is orthologous to Arabidopsis *AtCesA7* (irx3) gene associated with secondary cell wall synthesis. Gene.

[4] Kaur S, Dhugga KS, Gill K (2016). Novel structural and functional motifs in cellulose synthase (*CesA*) Genes of Bread Wheat (*Triticum aestivum*, L.). Plos One.

[5] Appenzeller L, Doblin M, Barreiro R (2004). Cellulose synthesis in maize: isolation and expression analysis of the cellulose synthase (*CesA*) gene family. Cellulose.

[6] Taylor NG, Laurie S, Turner SR (2001). Multiple cellulose synthase catalytic subunits are required for cellulose synthesis in Arabidopsis. Plant Cell.

[7] Taylor NG, Scheible WR, Cutler S (1999). The *irregular xylem3* locus of Arabidopsis encodes a cellulose synthase required for secondary cell wall synthesis. Plant Cell.

[8] Bhandari S, Fujino T, Thammanagowda S (2006). Xylem-specific and tension stress-responsive coexpression of *KORRIGAN* endoglucanase and three secondary wall-associated cellulose synthase genes in aspen trees. Planta..

[9] Yang C, Li D, Liu X (2014). OsMYB103L, an R2R3-MYB transcription factor, influences leaf rolling and mechanical strength in rice (*Oryza sativa* L.). BMC Plant Biol.

[10] Roberts JA, Evan D, Mcmanus MT (2018). Glycosyltransferases of the GT47 family. Annu Plant Rev.

[11] Zeng W, Lampugnani ER, Picard KL (2016). Asparagus *IRX9*, *IRX10*, and *IRX14A* are components of an active xylan backbone synthase complex that forms in the Golgi apparatus. Plant Physiol.

[12] Bonawitz ND, Chapple C (2010). The genetics of lignin biosynthesis: connecting genotype to phenotype. Annu Rev Genet.

[13] Silvia F, Montserrat C (2012). Altered lignin biosynthesis improves cellulosic bioethanol production in transgenic maize plants Down-regulated for Cinnamyl alcohol dehydrogenase. Mol Plant.

[14] Acker R, Vanholme R, Véronique S (2013). Lignin biosynthesis perturbations affect secondary cell wall composition and saccharification yield in *Arabidopsis thaliana*. Biotechnol Biofuels.

[15] Pilate G, Guiney E, Holt K (2020). Field and pulping performances of transgenic trees with altered lignification. Nat Biotechnol.

[16] Tu Y, Rochfort S, Liu Z (2020). Functional analyses of *Caffeic Acid O-Methyltransferase* and *Cinnamoyl-CoA-Reductase* genes from perennial ryegrass (*Lolium perenne*). Plant Cell.

[17] Wang Z, Li R, Xu J (2012). Sodium hydroxide pretreatment of genetically modified switchgrass for improved enzymatic release of sugars. Bioresour Technol.

[18] José C, Prinsen P, Rencoret J (2012). Structural characterization of the lignin in the cortex and pith of elephant grass (*Pennisetum purpureum*) stems. J Agric Food Chem.

[19] Nyambati E, Nyambati M, Sollenberger L (2003). Feed intake and lactation performance of dairy cows offered napiergrass supplemented with legume hay. Livest Prod Sci.

[20] Strezov V, Evans TJ, Hayman C (2008). Thermal conversion of elephant grass (*Pennisetum purpureum* Schum.) to bio-gas, bio-oil and charcoal. Bioresour Technol.

[21] Liu X, Shen Y, Lou L (2009). Copper tolerance of the biomass crops elephant grass (*Pennisetum purpureum* Schumach), Vetiver grass (*Vetiveria zizanioides*) and the upland reed (*Phragmites australis*) in soil culture. Biotechnol Adv.

[22] Somerville C, Youngs H, Taylor C (2010). Feedstocks for Lignocellulosic Biofuels. Science.

[23] Kawube G, Alicai T, Wanjala B (2015). Genetic diversity in Napier grass (*Pennisetum purpureum*) assessed by SSR markers. J Agric Sci.

[24] Bhandari P, Sukanya DH, Ramesh CR. Application of Isozyme Data in Fingerprinting napier grass (*Pennisetum purpureum* Schum.) for germplasm management. Genet Resour Crop Evol. 53 (2) (2006) 253–264; doi: 10.1007/s10722-004-6120-2.

[25] Harris K, Anderson W, Malik R (2010). Genetic relationships among napiergrass (*Pennisetum purpureum* Schum.) nursery accessions using AFLP markers. Plant Genet Resour.

[26] Zhou S, Chen J, Lai J, Yin G, Chen P (2019). Integrative analysis of metabolome and transcriptome reveals anthocyanins biosynthesis regulation in grass species *Pennisetum purpureu*m. Ind Crops Prod.

[27] Zhao J, Xia B, Meng Y, Yang Z, Pan L (2019). Transcriptome analysis to shed light on the molecular mechanisms of early responses to cadmium in roots and leaves of king grass (*Pennisetum americanum × P. purpureum*). Int J Mol Sci.

[28] Jakob K, Zhou F, Paterson AH (2009). Genetic improvement of C4 grasses as cellulosic biofuel feedstocks. Vitro Cell Dev Biol Plant.

[29] Dixon R, Sewalt V, Howles P (1996). Genetic manipulation of the phenylpropanoid pathway in transgenic tobacco: new fundamental insights and prospects for crop improvement. Biotechnol Biotechnol Equip.

[30] Huang G, Zeng Y, Wei L, Yao Y. Comparative transcriptome analysis of mulberry reveals anthocyanin biosynthesis mechanisms in black (*Morus atropurpurea* Roxb.) and white (*Morus alba* L.) fruit genotypes. BMC Plant Biol. 2020;20(1). 10.1186/s12870-020-02486-1. 10.1186/s12870-020-02486-1 PMC7301479 32552771

[31] Kanno M, Kijima A. Quantitative and Qualitative Evaluation on the Color Variation of the Japanese Sea Cucumber *Stichopus japonicus*. Aquaculture Sci. 50 (1) (2002) 63–69; doi: 10.11233/aquaculturesci1953.50.63.

[32] Douglas CJ (1996). Phenylpropanoid metabolism and lignin biosynthesis: from weeds to trees. Trends Plant Sci.

[33] Kleczkowski LA (1994). Glucose activation and metabolism through UDP-glucose pyrophosphorylase in plants. Phytochemistry.

[34] Martin LK, Haigler CH (2004). Cool temperature hinders flux from glucose to sucrose during cellulose synthesis in secondary wall stage cotton fibers. Cellulose.

[35] John W, Downton S, Hawker JS (1973). Enzymes of starch and sucrose metabolism in *Zea mays* leaves. Phytochemistry.

[36] Lu X, Li C, Wang X, Zhang W, Xia T (2019). Enzymatic sugar production from elephant grass and reed straw through pretreatments and hydrolysis with addition of thioredoxin-his-S. Biotechnol Biofuels.

[37] Xu B, Luis L, Escamilla T, Sathitsuksanoh N (2011). Silencing of 4-coumarate: coenzyme a ligase in switchgrass leads to reduced lignin content and improved fermentable sugar yields for biofuel production. New Phytol.

[38] Paul H, Sameer A, Masoud JW. Overexpression of L-phenylalanine ammonia-lyase and cinnamate 4-hydroxylase in tobacco cell suspension cultures. Plant Biotechnology and In Vitro Biology in the 21st Century. 20 (1999) 297–301; doi: 10.1007/978-94-011-4661-6_69.

[39] Boerjan W, Ralph J, Baucher M (1999). Lignin biosynthesis. Annu Rev Plant Biol.

[40] Qin L, Qin X, Chen Y, Zeng W (2016). The cotton β-galactosyltransferase 1 (*GalT1*) that galactosylates arabinogalactan-proteins participates in controlling fiber development. Plant J.

[41] Lu X, Zheng X, Li X (2016). Adsorption and mechanism of cellulase enzymes onto lignin isolated from corn Stover pretreated with liquid hot water. Biotechnol Biofuels.

[42] Du J, Cao Y, Liu G (2017). Identifying and overcoming the effect of mass transfer limitation on decreased yield in enzymatic hydrolysis of lignocellulose at high solid concentrations. Bioresour Technol.

[43] Sluiter A, Hanes B, Ruiz R, Scarlata C, Sluiter J. Determination of structural carbohydrates and lignin in biomass. National Renewable Energy Laboratory (NREL) Laboratory Analytical Procedures (LAP) for standard biomass analysis. 2007;25:275–301.

[44] Liu Y, Muhammad R, Yan L, Zeng Y, Jiang C (2019). Boron and calcium deficiency disturbing the growth of trifoliate rootstock seedlings (Poncirus trifoliate L.) by changing root architecture and cell wall. Plant Physiol Biochem.

[45] Jiang C (2019). Efficient extraction of RNA from various Camellia species rich in secondary metabolites for deep transcriptome sequencing and gene expression analysis. Afr J Biotechnol.

[46] Grabher R, Manfred G (2011). Full-length transcriptome assembly from RNA-Seq data without a reference genome. Nat Biotechnol.

[47] Limin F, Beifang N, Zhu Z (2012). CD-HIT: accelerated for clustering the next generation sequencing data. Bioinformatics.

[48] Patro R (2017). Salmon provides fast and bias-aware quantification of transcript expression. Nat Methods.

[49] Davidson NM, Oshlack A (2014). Corset: enabling differential gene expression analysis for de novoassembled transcriptomes. Genome Biol.

[50] Young MD, Wakefield MJ, Smyth GK (2010). Gene ontology analysis for RNA-seq: accounting for selection bias. Genome Biol.

[51] Zhang B, Horvath S (2005). A general framework for weighted gene coexpression network analysis. Stat Appl Genet Mol Biol.

[52] Langfelder P, Horvath S (2008). WGCNA: an R package for weighted correlation network analysis. BMC Bioinformatics.

