# Supplementary figures and images for: Tissue-specific Transcriptome analysis reveals lignocellulose synthesis regulation in elephant grass (Pennisetum purpureum Schum)
Source: BMC Plant Biol. 2020 Nov 19;20:528. doi: 10.1186/s12870-020-02735-3 (PMC7678330; doi:10.1186/s12870-020-02735-3)

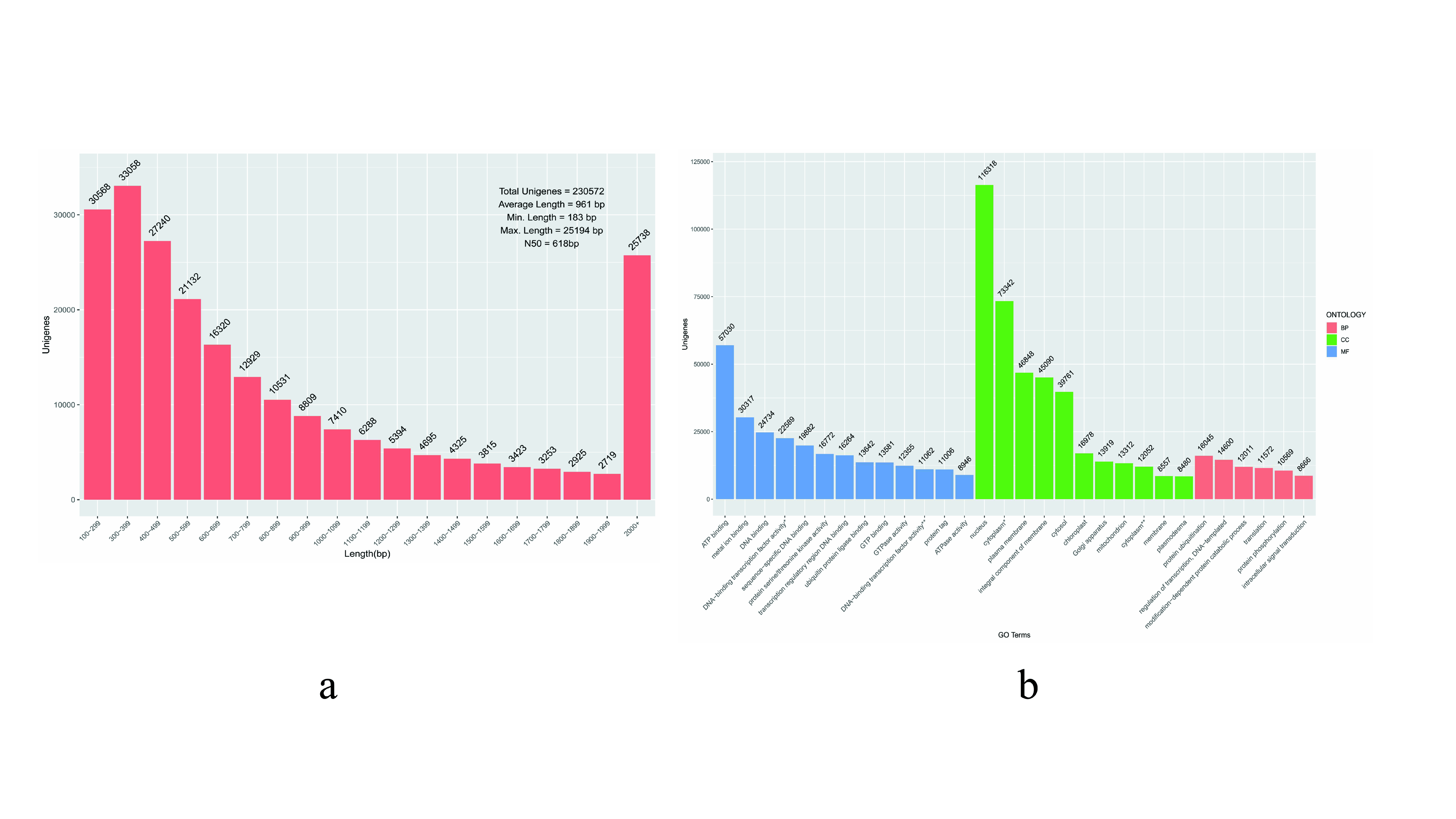

Supplement: Supplementary file 2 — Additional file 2: Fig. S2. Distribution and annotation statistics of assembled genes. (a) Length distribution and summary statistics of assembled genes. (b) In each of the three GO categories, the unigene distribution representing the most extensive level 3 gene ontology (GO). biological processes (BP), cellular components (CC) and molecular functions (MF). [file 12870_2020_2735_MOESM2_ESM.tif]

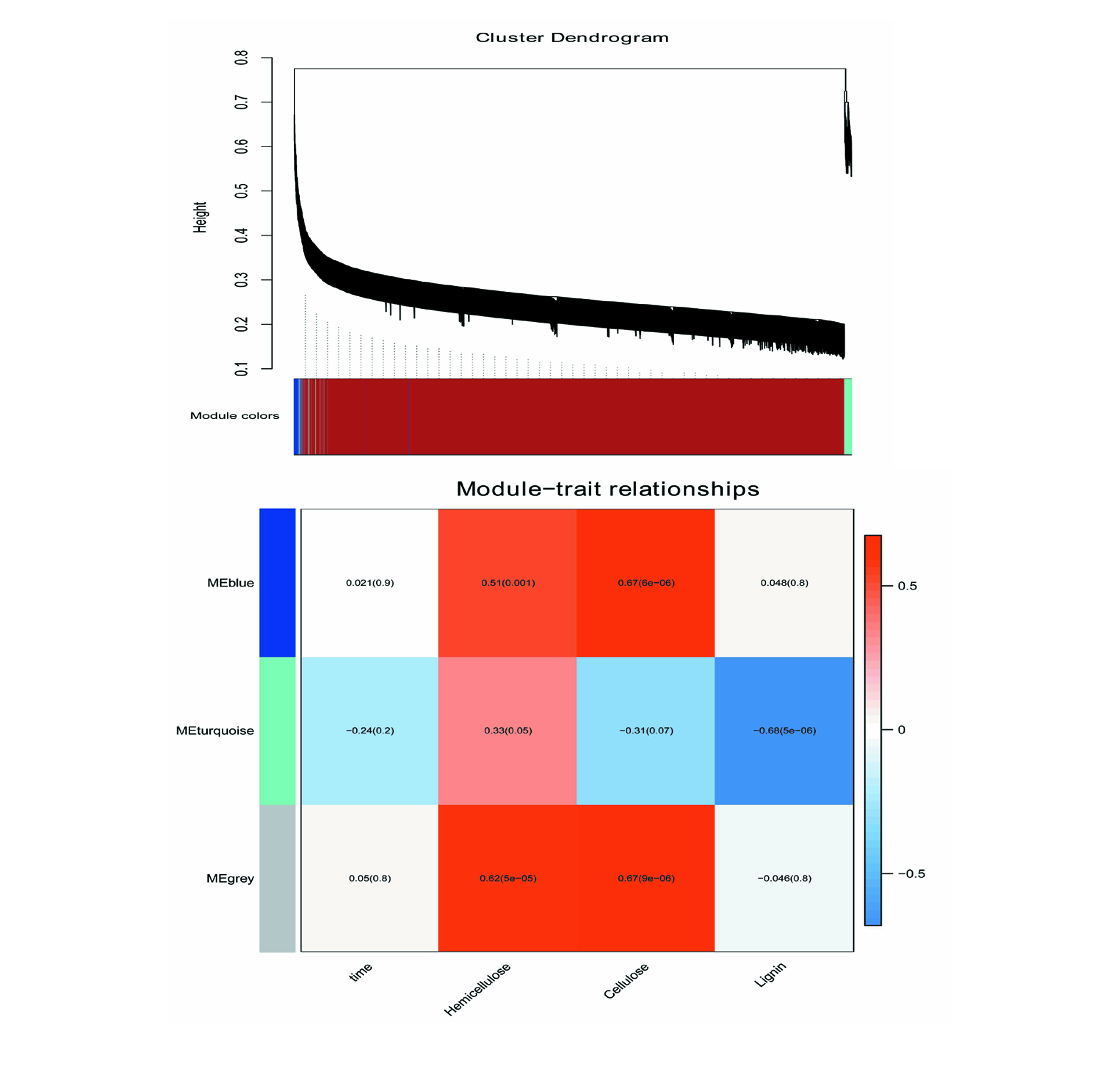

Supplement: Supplementary file 4 — Additional file 4: Fig. S4. 3852 DEG sets co-expressed at three developmental stages of T1, T2 and T3 were analyzed by WGCNA. [file 12870_2020_2735_MOESM4_ESM.tif]

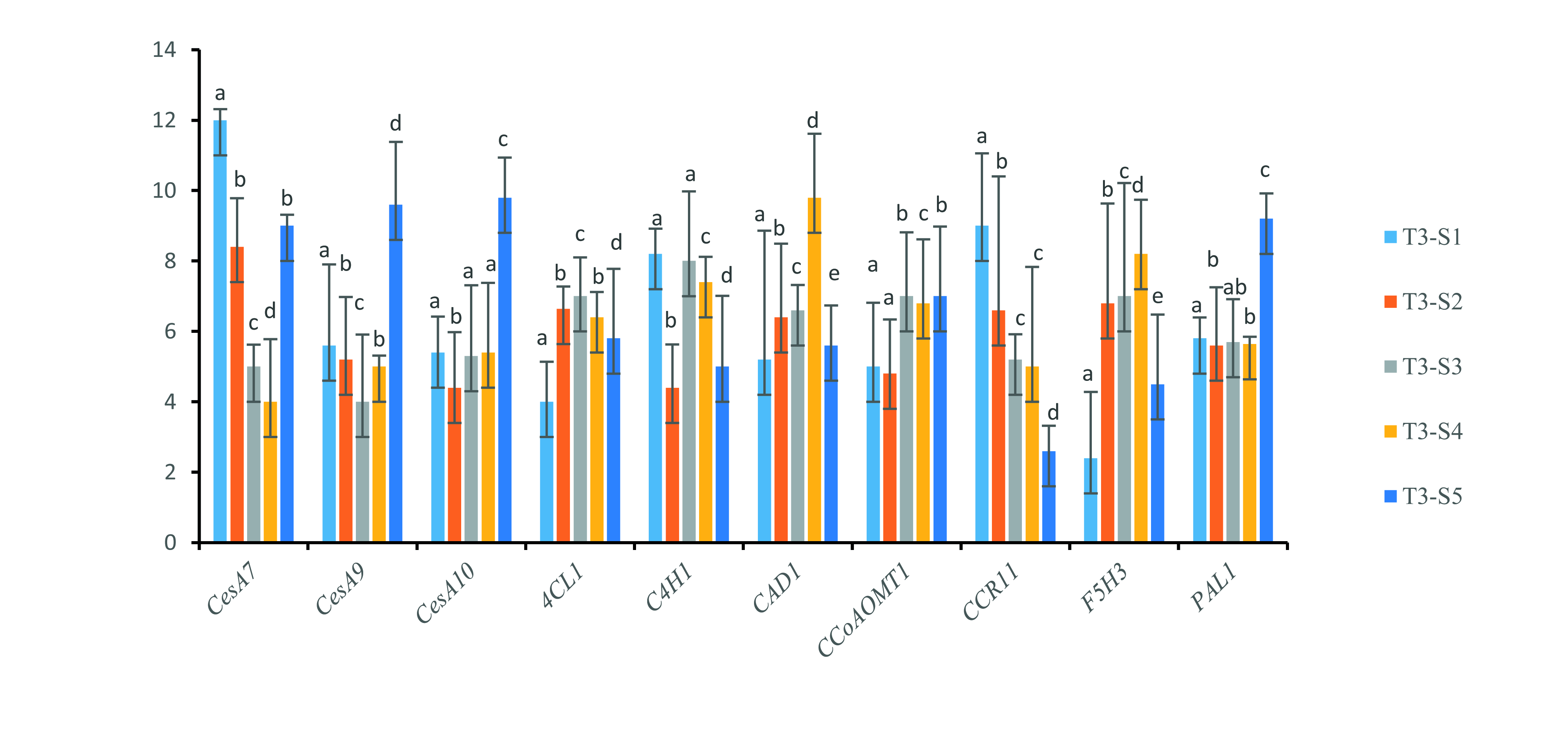

Supplement: Supplementary file 5 — Additional file 5: Fig. S5. RT-PCR analysis of synthetic genes related to cellulose and lignin synthesis in elephant grass stems. Different letters indicate statistically significant differences (ANOVA, Duncan < 0.05). [file 12870_2020_2735_MOESM5_ESM.tif]
