# Supplementary material for: Tissue-specific Transcriptome analysis reveals lignocellulose synthesis regulation in elephant grass (Pennisetum purpureum Schum)
Source: BMC Plant Biol. 2020 Nov 19;20:528. doi: 10.1186/s12870-020-02735-3 (PMC7678330; doi:10.1186/s12870-020-02735-3)
Supplement: Supplementary file 10 — Additional file 10: Table S5. RT-PCR primer list. [file 12870_2020_2735_MOESM10_ESM.rtf]

Table 3 Primer list used in qRT-PCR analysis 
Transcript ID 	Name 	Forward (5′→3′)	Reverse (5′→3′)	Tm (◦C)	Amplicon Length (bp) 	
Cluster-79233.3	C4H1	AACCTAGTCGTCGTCTCCTCACC	CAATGCTCGCCGTAAACAGTGAAC	63.3	149	
Cluster-18695.4188	CESA7	ACTGGAATCCTGGAGCTGAGATGG	GCGGATGTGCCACCAATGACC	61.7	89	
Cluster-82155.4	F5H3	GGTGGCTCGACAAGTCCTTCAAG	TCCGCTCGGTCGTAAGTCAGATAG	59.5	93	
Cluster-18695.4138	CESA9	TTCAGCACAGGCGAGATCAATGG	TACGGTCCTTCCAAGCAACACTTC	59.4	80	
Cluster-18695.23515	CCR11	CGGCAAGATGGTGGCGGAAC	GCTGTAACGGCGGTCCAAGAAC	59.2	104	
Cluster-18695.54189	4CL1	GAATCCGTGCCAATCCCTGAAGG	 TCGTGCCAGAGGAGTAAGGTAGTG	59.3	136	
Cluster-62152.8	CAD1	CTGTGCTGGAATCACGGTTTATGC	AAGACCACCTAGCCCGATCACC	59.1	91	
Cluster-82361.1	CCoAOMT1	GTCACATGCCTCGCCTTGCC	GCTACTCCTTGTCCTTGGGTGTTG	60.3	111	
Cluster-18695.9383	PAL1	GCCACCTACATCTACGCCAACG	TACGAGGACCAAGCAAGCAAAGC	59.3	109	
Cluster-18695.4033	CESA10	TGGGTCATTGGAGGAGTCTCTTCG	TGTGACTGTGAAGTTCGTGCTGAC	62.6	93	
